# Clinicopathological spectrum of non-sinonasal intestinal-type adenocarcinomas of the head and neck: Systematic review of case reports, case series, and cross-sectional studies

**DOI:** 10.4317/medoral.27770

**Published:** 2025-11-22

**Authors:** Gustavo de-S Vieira, Moisés W A Gonçalves, Priscila C Tincani, Alfio J Tincani, Carlos T Chone, Arthur Antolini-Tavares, Albina Altemani, Fernanda V Mariano

**Affiliations:** 1 Department of Oral Diagnosis, Piracicaba Dental School, Universidade Estadual de Campinas (UNICAMP), Piracicaba, SP, Brazil; 2 Department of Pathology, Faculty of Medical Sciences, Universidade Estadual de Campinas (UNICAMP), Campinas, SP, Brazil; 3 Department of Surgery, Division of Head and Neck Surgery, Faculty of Medical Sciences, Universidade Estadual de Campinas (UNICAMP), Campinas, SP, Brazil; 4 Department of Ophthalmology and Otorhinolaryngology, Faculty of Medical Sciences, Universidade Estadual de Campinas (UNICAMP), Campinas, SP, Brazil

## Abstract

**Background:**

Intestinal-type adenocarcinomas (ITACs) most often arise in the sinonasal tract, typically associated with occupational exposures, but they rarely occur in other head and neck sites. When present in extra-sinonasal regions, their clinicopathological and molecular characteristics remain poorly understood. This systematic review aimed to clarify the clinicopathological, immunohistochemical, and molecular features of non-sinonasal intestinal-type adenocarcinomas (ITACs) of the head and neck.

**Material and Methods:**

This review was conducted according to PRISMA 2020 guidelines and registered in PROSPERO (CRD42022309841). Two reviewers independently screened, extracted data, and assessed risk of bias using the Joanna Briggs Institute tools. Sources included PubMed/MEDLINE, Scopus, Embase, Web of Science, Google Scholar, and OpenGrey. A comprehensive search identified 1,376 records. After applying eligibility criteria, 26 studies comprising 37 cases were included. Data on clinical, histological, immunophenotypic, molecular, and prognostic features were analyzed.

**Results:**

Most patients were male (73%), with a mean age of 57.9 years. The oral cavity, particularly the mobile tongue (51.4%), was the most commonly affected site. Histologically, colonic (59.5%) and mucinous (56.8%) architectures were the most frequent microscopic patterns presented. Immunohistochemistry frequently showed positivity for CK7, CK20, and CDX2, while SATB2, MUC1, and MUC5AC had variable expression. Mismatch repair proteins were intact in all cases. Molecular findings included mutations in MLL3, TP53, EGFR, and AKT1, and upregulation of PAX1, MUC5B, and EMT-related genes, suggesting a distinct profile from sinonasal ITACs. Surgical resection, often with adjuvant therapy, was the main treatment. Tumors were aggressive, with metastases being present in 35.1% and disease-specific mortality in 24.3%.

**Conclusions:**

Non-sinonasal ITACs are rare, aggressive malignancies requiring accurate diagnosis and further molecular investigation to improve management and outcomes.

## Introduction

Intestinal-type adenocarcinoma (ITAC) is a rare malignant epithelial neoplasm characterized by histological and immunophenotypic features that closely resemble colorectal adenocarcinoma (1 , 2). In the head and neck, sinonasal ITAC is a well-defined subtype within the classification of sinonasal adenocarcinomas, which are broadly divided into intestinal-type and non-intestinal-type, and is frequently linked to occupational exposure to inhaled organic dusts, especially from wood and leather, highlighting a strong predilection for this anatomic region (1 , 3 , 4).

Typically, ITAC affecting the head and neck presents as aggressive tumors that invades and destroys adjacent tissues, predominantly arising in the ethmoid sinuses, and occasionally found in the nasal cavity and maxillary sinus (3). However, primary occurrences in extra-sinonasal locations, such as the major salivary glands or the oral cavity are exceedingly rare, and the histogenesis in these contexts remains poorly understood (5 - 8). Clinically, it is an aggressive tumor with high rates of local recurrence, less frequent distant metastases, and overall poor prognosis. Surgery remains the primary modality of treatment (2).

ITAC exhibits atypical glandular structures, nuclear stratification, prominent 'dirty' necrosis, and an infiltrative growth pattern, closely resembling the morphological features of colorectal adenocarcinoma on hematoxylin and eosin (H&amp;E) staining. Immunophenotypic markers in addition to these aspects are fundamental for a correct diagnosis, including CK20 and CDX2, confirming the lower-gastrointestinal tract phenotype (1 , 2 , 7 , 9). With all the microscopic features characteristic and necessary for the successful detection of an ITAC in the head and neck region, a systematic search for primary lesions in the gastrointestinal tract is necessary to confirm this diagnosis (7).

While sinonasal ITACs have been the subject of several molecular studies, including transcriptomic and mutational profiling, similar investigations in non-sinonasal counterparts are limited to isolated case reports and small case series (2 , 10 - 12). The absence of large-scale analyses hinders both diagnostic standardization and therapeutic development. Given the growing availability of molecular diagnostics and targeted therapies, a better understanding of the clinicopathological and genetic features of non-sinonasal ITACs is urgently needed.

The present study aims to provide a comprehensive synthesis of published cases of non-sinonasal ITACs of the head and neck. By integrating clinical, histopathological, immunohistochemical, and molecular data, we seek to define the distinguishing characteristics of this rare entity and to explore its potential relationship with other adenocarcinoma subtypes of intestinal and salivary gland origin. Therefore, we structured this review to address the following PICO-based research question: "What are the clinicopathological, therapeutic, and outcome characteristics of non-sinonasal Intestinal-type adenocarcinomas of the head and neck?".

## Material and Methods

Study protocol

This systematic review targeted studies that report human cases of non-sinonasal ITACs of the head and neck, and was carried out following the Preferred Reporting Items for Systematic Reviews and Meta-Analyses (PRISMA, 2020) guidelines (13). The review was registered in the International Prospective Register of Systematic Reviews (PROSPERO) with the application identifier (CRD42022309841).

According to PECO model, we established the following as study elements: P (population): Patients with non-sinonasal ITACs of the head and neck; E (exposure): Clinical and pathological characterization (e.g., histopathological features, immunohistochemical markers, molecular profiles, treatment approaches); C (comparator): Not applicable; O (outcomes): Prognosis and clinical outcomes (e.g., recurrence, survival). In this context, we formulate the following question: "What are the clinicopathological, therapeutic, and outcome characteristics of non-sinonasal intestinal-type adenocarcinomas of the head and neck?".

Search Strategy

The target period set was up to April 20, 2025, in which the studies were selected using the following search strategies according to the supplementary material 1 (http://www.medicina.oral.com/carpeta/suppl1_27770), using the Medline/PubMed, Scopus, Embase, Web of Science as main databases, as well as the Google Scholar and OpenGrey (DANS EASY archive) databases targeting grey literature. in accordance with PRISMA 2020 recommendations.

Eligibility criteria

Studies were included if they were original articles (case reports, case series, and cross-sectional studies) describing non-sinonasal ITACs of the head and neck that reported neoplastic carcinomatous epithelium that mirrors neoplastic intestinal mucosa and its morphological variants, and reported at least one of the following domains: Clinical presentation, immunohistochemical, treatment, or outcome aspects. Only studies published in English and which mentioned the exclusion of a metastatic intestinal adenocarcinoma were considered eligible. Exclusion criteria comprised review articles, editorials, letters to the editor, conference abstracts, studies focusing exclusively on sinonasal tumors, and studies lacking relevant clinical and pathological information that could confirm the diagnosis. The selection process was conducted independently by two reviewers (G.S.V. and M.W.A.G.) who screened titles and abstracts for eligibility. Full-text review was performed for studies meeting inclusion criteria or where eligibility was unclear. Discrepancies were resolved by consensus or consultation with a third reviewer (F.V.M.).

Data extraction assessment

Data extraction was performed independently by two reviewers (G.S.V. and M.W.A.G.) using a standardized form. Information collected included patient demographics (age, gender, country, associated factors), clinical presentation (tumor location, symptoms, and size), histopathological, immunohistochemical and molecular features, treatment strategies (surgery, radiotherapy, chemotherapy), and clinical outcomes (follow-up time, recurrence, metastasis, survival, and death). Disagreements between reviewers were resolved through discussion. The methodological quality of the included studies was appraised with the Joanna Briggs Institute critical appraisal tools, according to study design.

Data analysis

Data synthesis was conducted using descriptive statistics and survival analysis. Missing information was coded as 'not specified' and reported in the quantitative summaries. Given the rarity of these tumors and the heterogeneity of study designs, meta-analysis was not feasible. Consequently, no pooled estimates were generated; instead, outcomes were narratively compared, and patterns across histological subtypes and anatomical sites were qualitatively summarized. No subgroup or sensitivity analyses were planned, which represents an additional limitation of the review. Survival analysis was based on disease-specific survival, with diagnosis (when not treated) or end of treatment as the starting point and the longest follow-up/outcome period as the endpoint. For this analysis, the Kaplan-Meier curve was used, and the statistical value obtained according to the Log-rank test, which was significant when p&lt;0.05.

Risk of bias assessment

The risk of bias in individual studies was independently assessed by two authors using the Joanna Briggs Institute critical appraisal tool for each study (14). The risk of bias was classified as high if the study reached up to 49% "yes"; moderate if the study reached 50% to 69% "yes"; and low if the study reached at least 70% "yes". Disagreements were resolved first by discussion and then by consulting a senior author (F.V.M.).

## Results

Search and selection of studies

**Figure 1:**
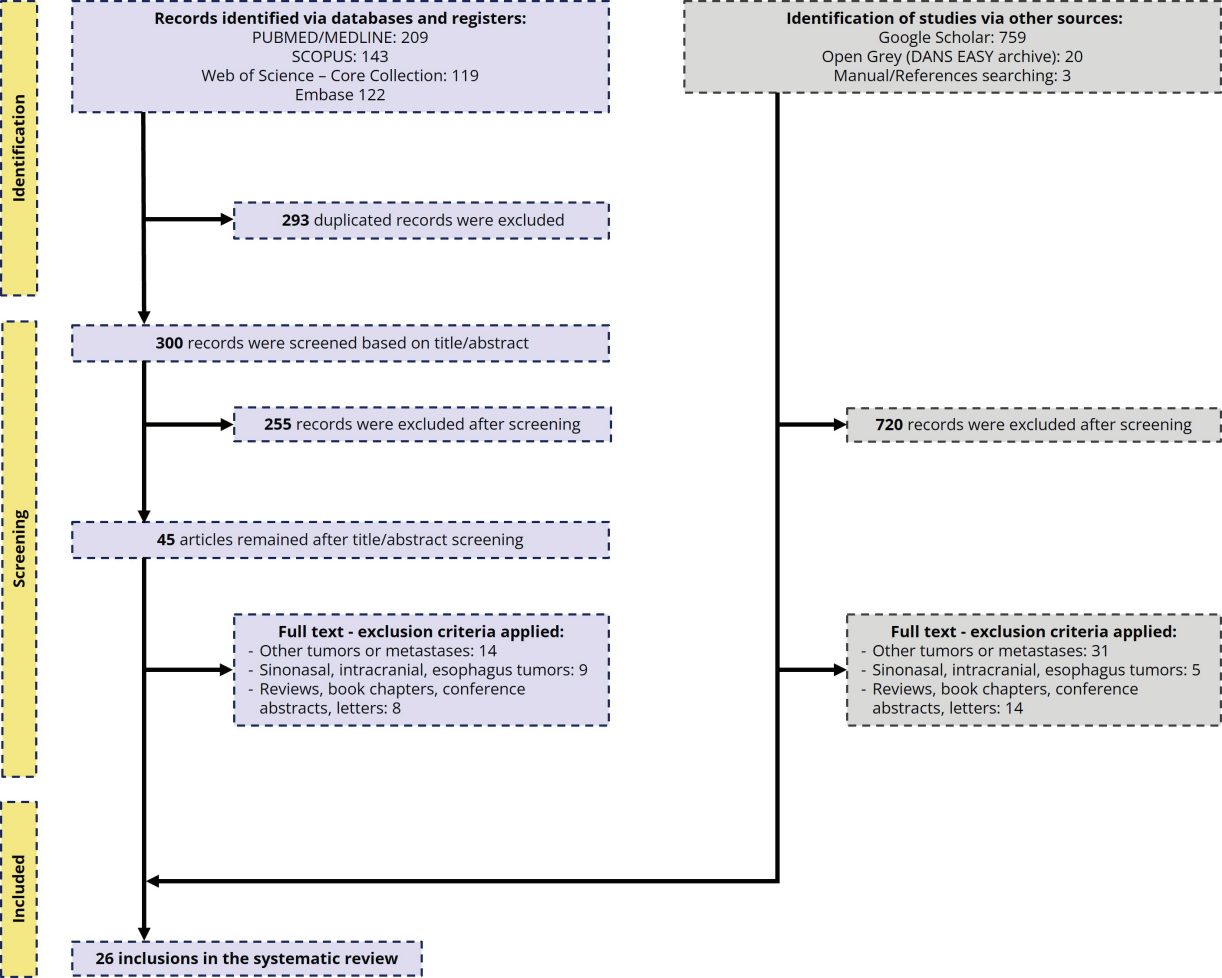
Flowchart of article selection according to PRISMA 2020 guidelines.

A total of 1,376 records were identified: 593 from major scientific databases (PubMed/MEDLINE, Scopus, Web of Science, Embase) and 782 from grey literature sources, including Google Scholar (n=759), OpenGrey (n=20), and manual reference searches (n=3). After removing 293 duplicates, 300 records underwent title and abstract screening, resulting in the exclusion of 255 records. The remaining 45 articles were assessed in full, with 31 excluded based on predefined criteria such as non-relevant tumor types, anatomical site (e.g., sinonasal, intracranial, esophageal), or publication type (e.g., reviews, conference abstracts, book chapters, letters). Applying the same criteria to the grey literature findings, a total of 770 publications were excluded in the whole process. In total, 26 studies fulfilled the eligibility criteria and were included in the systematic review (Figure 1).

1

Clinical characteristics

A total of 37 cases of non-sinonasal ITACs of the head and neck were analyzed (Table 1). The majority of cases originated from the United States (43.2%), followed by China (18.9%), and India (10.8%), with individual cases reported in Brazil, Germany, Canada, Japan, South Africa, South Korea, Spain, and the United Kingdom. There was a marked male predominance (73%), and the mean patient age was 57.94 years (±14.50), with the highest incidence observed during the sixth and seventh decades of life (29.7% each). Among the possible risk factors, the most frequently reported was smoking (27%), but a significant percentage of the publications did not report this (62.2%). Clinically, the oral cavity was the most frequently involved anatomical site (51.4%), particularly the mobile tongue (29.7%), followed by the base of tongue in the oropharynx (18.9%) and major salivary glands (10.8%), especially the submandibular gland (8.1%). Other less common locations included the nasopharynx, hypopharynx, larynx, ear complex, facial skin, and ectopic salivary glands. Swelling was the most common presenting symptom (67.6%), followed by dysphagia (21.6%) and pain (10.8%). The mean tumor size was 3.99 cm (±2.47), although size was not reported in 43.2% of cases. Associated lymphadenopathy was reported in 35.1% of cases. The mean duration of symptoms before diagnosis was 34.31 months (±118.87), although more than half of the cases (56.8%) did not specify this information.

Table 1

Histopathological characteristics

As precursor lesions, the existence of a foregut duplication cyst in 3 cases (8.1%), a congenital cystic teratoma (2.7%), and a gastric heterotopia in the lingual dorsum (2.7%) was mentioned. Despite being named heterogeneously among the published articles, the reported cases could be classified according to the 5th World Health Organization Classification of Head and Neck Tumors, presenting the following morphological patterns: Villous/colonic (59.5%), mucinous (56.8%), papillary/tubulopapillary (27%), signet-ring (13.5%), cribriform (8.1%), or not specified (8.1%). Nearly half of the cases (45.9%) showed moderate differentiation. Additional notable cyto-morphological features included the presence of goblet cells (54.1%), infiltrative/invasive growth (54.1%), high mitotic activity (48.6%), necrosis (43.2%), cystic/multi-cystic spaces (27%), and squamous or neuroendocrine differentiations (2.7%) were frequently observed. Perineural or lymph-vascular invasions were not frequently, reported in 10.8% and 8.1% of cases, respectively (Table 1).

Immunohistochemical profile

Immunohistochemical analysis demonstrated that CK7 (79.4%; 27/34), CK20 (70%; 21/30) and CDX2 (58.3%; 14/24) were frequently positive, corroborating the intestinal-like differentiation of these head and neck tumors (Table 2). Despite being rarely reported, SATB2 was positive in all cases when mentioned. High Ki-67 index ranged from 60% to 90% when mentioned, indicative of the high proliferative nature of these neoplasms. Additional positive immunomarkers included AE1/AE3, -catenin, MUC1, CEA, EMA, and synaptophysin. Negative staining for p63, TTF-1, S100, and MUC2 was also common, assisting in the exclusion of other differential diagnoses such as salivary gland tumors and pulmonary or thyroid primaries. Mismatch repair (MMR) genes markers (hMLH1, hMSH2, hMSH6, hPMS-2) used for the evaluation of microsatellite instability were always positive, demonstrating head and neck ITACs affecting non-sinonasal regions are not affected by genetic instability or MMR deficiency.

Table 2

Genetic alterations

Only three papers reported genetic features, including genomic and transcriptomic data (Table 3). Genomic studies identified recurrent somatic mutations in MLL3, MAP3K4, TP53, and EGFR genes. In addition, AKT1 E17K hotspot mutations were observed in a subset of cases reporting mucinous adenocarcinoma features. Transcriptomic analyses demonstrated upregulation of PAX1, NKX3-1, BPIFB6, BPIFB2, and MUC5B genes. Furthermore, a specific TP53 missense mutation (Leu194His) was identified in one case, while no mutations were detected in the KRAS, NRAS, BRAF, or PIK3CA genes. These findings highlight molecular pathways possibly contributing to tumorigenesis and offer potential targets for future therapeutic interventions.

Table 3

Treatment and outcomes

**Figure 2:**
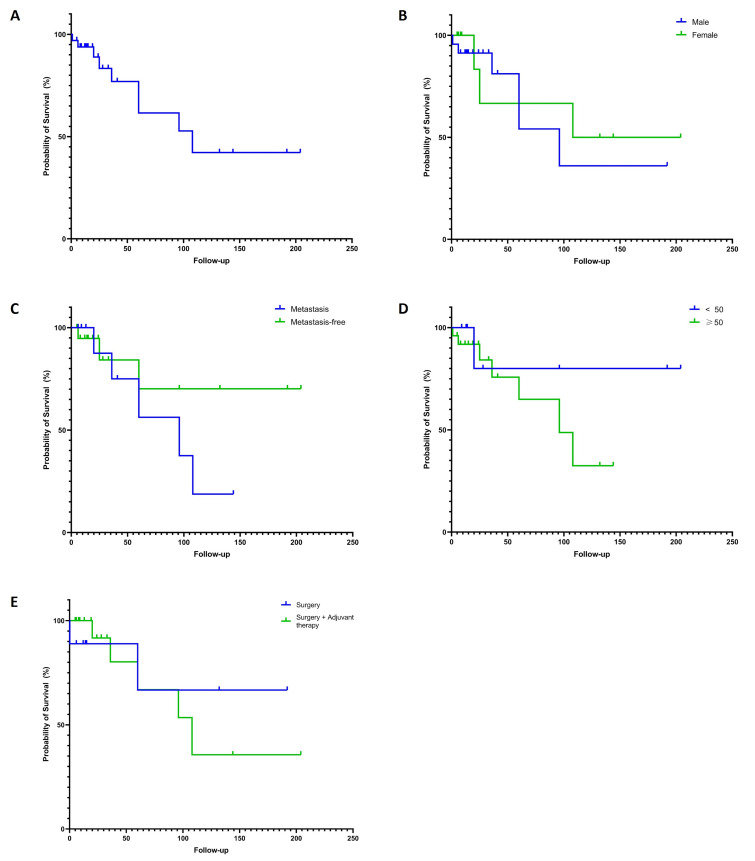
Disease-specific survival (DSS) rates of the non-sinonasal intestinal-type adenocarcinomas of the head and neck. General DSS (A) and DSS stratified according to gender (B), age (C), presence of metastasis (D), and combination of adjuvant therapies with surgery.

Most patients were managed with multimodal therapeutic strategies (Table 1). Surgery alone (24.3%) or combined with adjuvant therapies (chemotherapy, radiotherapy, and/or immunotherapy; 54.1%) was the mainstay of treatment. Surgery combined with chemotherapy and radiotherapy was the most frequent approach (27%), while 10.8% received chemoradiotherapy without surgical intervention. Local recurrence was observed in 8.1% of patients, with a mean recurrence interval of 12 months. Regional and/or distant metastases occurred in 35.1% of cases, predominantly affecting lymph nodes (32.4%) and lungs (16.2%). Other metastatic sites included the brain, ovary, skin, and submandibular gland. At the time of last follow-up, 54.1% of patients were disease-free, 16.2% were alive with disease, and 24.3% had succumbed to the disease, with a mean time to death of 38.17 months (±45.47). The 5-year and 10-year disease-specific survivals were 61.54% and 42.20%, respectively (Figure 2A). No significant differences in survival rates were observed by gender (p=0.6105), age (&lt;50 vs. 50; p=0.2820), presence of metastasis (p=0.2479), or the use of adjuvant therapy combined with surgery (p=0.7497), according to the Log-rank test (Figure 2B-2E). The mean follow-up period was 46.12 months (±54.91).

2

Risk of bias within studies

The Joanna Briggs Institute risk of bias assessment was conducted for case reports, case series, and cross-sectional studies. In the case series, in general, all studies were classified as having a low risk of bias. Nevertheless, two studies (50%) demonstrated a high risk related to patient inclusion criteria, and all studies (100%) presented a high risk in the domain of statistical analysis. The single cross-sectional study included was also assessed as having a low risk of bias. For case reports, in general, 20 studies were classified as having a low risk of bias, while only one study was considered high risk. However, 9 studies (42.9%) presented an uncertain risk with regard to diagnostic methods, mainly due to limitations in immunohistochemical analysis, and 13 studies (61.9%) presented a high risk in the reporting of adverse events. Supplementary table 2 (http://www.medicina.oral.com/carpeta/suppl2_27770) provides a detailed breakdown of the risk of bias assessment for each included study across individual domains of the JBI tool, as recommended by PRISMA 2020.

## Discussion

This study consolidates the most extensive set of clinicopathological, immunohistochemical, and molecular data to date on non-sinonasal ITACs of the head and neck. The findings provide important insights into the biological behavior, diagnosis, and management of these rare tumors.

The patient cohort showed a clear male predominance and a mean age of approximately 58 years, with most cases diagnosed in the sixth and seventh decades of life. This trend maintained the panorama presented by the sinonasal region (10 , 15 - 17). Wood dust, the main exposure factor in sinonasal cases, (18) was absent from most reports that mentioned associated factors (6); smoking history was cited in 27%, (19 - 28) though exposure factors were often not addressed. A strong predilection for the oral cavity, particularly the mobile tongue, (2 , 20 - 22 , 24 , 28 - 33) was evident, although involvement of the oropharynx (tongue base) (2 , 5 , 6 , 19 , 23 , 34) also being expressive, with the lingual structure alone accounting for 48.6% of the sample. Tumors frequently presented as swelling of variable sizes at the time of the diagnosis, highlighting a pattern of clinical detection of up to 6 months, with usual regional lymphadenopathy.

The origin of intestinal-type adenocarcinomas in the head and neck remains uncertain (4 , 19). It is believed that it should emerge from mucosal or salivary epithelium with aberrant intestinal differentiation, (19) or from precursor lesions that can present gastrointestinal phenotype, such as foregut duplication cysts, teratomas, or gastric heterotopia, as suggested in prior reports (21 , 28 , 31 , 34 , 35). The current 5th World Health Organization classification of head and neck tumors classifies the ITAC as a variant of salivary carcinoma, not otherwise specified, with molecular findings supporting a primary origin rather than metastasis from the gastrointestinal tract (1). Microscopically, the tumors exhibited colonic and mucinous phenotypes most frequently, followed by papillary, showing a classification aligned with that initially proposed by Barnes in 1986 for sinonasal cases, (36) with the subtype frequencies varying in this location (15 , 36). The final microscopic diagnosis was reflected in the evidence of an intestinal-type phenotype, on its most prominent morphological subtype, in the classification as a diagnosis of exclusion, as referred to by the WHO, (1) or even drawing attention to the presence of concurrent areas of distinct carcinomatous differentiation (neuroendocrine or squamous) (26 , 31). Notably, some tumors demonstrated histological diversity, suggesting potential heterogeneity in pathogenesis or tumor progression pathways (15 , 36).

The immunohistochemical profile strongly supported intestinal differentiation. Expression of CK20 and CDX2 was frequent, a pattern reminiscent of colorectal carcinomas rather than conventional salivary gland tumors, while CK7 expression corroborates with a primary head and neck tumor (37). SATB2 positivity, when present, reinforced the diagnosis of intestinal-like adenocarcinoma (38). Negative staining for markers such as p63, S100, and TTF-1 proved essential in excluding other primary malignancies, particularly thyroid, pulmonary, and salivary gland carcinomas. Collectively, these findings highlight the importance of an extended immunohistochemical panel for accurate diagnosis, especially in small biopsy specimens. In colorectal carcinoma, loss of mismatch repair proteins (MLH1, MSH2, MSH6, PMS2) by immunohistochemistry indicates microsatellite instability, which has diagnostic and therapeutic implications, including possible Lynch syndrome and eligibility for immunotherapy (39). When evaluated in ITAC, these markers were always positive, demonstrating the absence of instability among this tumor (5 , 24 , 26 , 32). In our sample, ITACs often show differential mucin expression, with frequent positivity for MUC1 and MUC5AC and variable or absent expression of MUC2. In colorectal cancer, MUC1 and MUC5AC are commonly upregulated comparing to normal mucosa, while MUC2 expression is typically restricted to mucinous subtypes; notably, MUC1 overexpression has been strongly associated with poor prognostic outcomes (40 , 41).

A recent molecular study has highlighted key distinctions between sinonasal and non-sinonasal ITACs of the head and neck (2). This multi-omics joined transcriptome and exome analysis demonstrated that non-sinonasal ITACs exhibit a markedly higher number of differentially expressed genes compared to sinonasal counterparts (2909 vs. 410), suggesting greater molecular heterogeneity. In addition, non-sinonasal tumors showed upregulation of genes involved in epithelial-mesenchymal transition, immune modulation, and anti-apoptotic signaling (e.g., FOXA3, SPDEF, HNF1B, EpCAM, TOX3, and TNFSF11), while sinonasal cases overexpressed developmental genes like LEFTY1, OLFM4, ZFP57, ALX, and PDX1. Notably, the chromatin remodeling gene MLL3 (KMT2C) exhibited frequent loss-of-function mutations across both subtypes, pointing to epigenetic deregulation as a shared oncogenic mechanism (2). Frequent mutations affecting the TP53 gene were also observed (2 , 32). Rooper et al. (2021) (42) reported recurrent AKT1 E17K mutations in salivary mucinous adenocarcinomas, a modification also found in four out of six cases (66.7%) of papillary adenocarcinoma with intestinal-type features affecting the minor salivary glands (30). These findings corroborate previous evidences that some ITACs may also have a salivary gland origin, especially those with a mucinous phenotype.

The current treatment scenario, largely based on surgery combined with adjuvant therapies, seems insufficient to control disease progression in a significant subset of patients, given the considerable number combining the rates of recurrence, metastasis, disease progression and death from disease (45.9%; n=17) (2 , 5 , 6 , 8 , 19 , 22 , 24 , 25 , 27 , 30 , 32 , 33 , 43 , 44). The substantial rates observed in this series call for developing new therapeutic strategies. Lymphatic and distant spread rates were considerable, with metastases most commonly involving lymph nodes and lungs (2 , 5 , 6 , 8 , 19 , 22 , 25 , 27 , 32 , 33 , 43). The high frequency of metastases or locoregional recurrences mentioned point to the need for a radical regional and cervical lymphadenectomy during the surgical management of these neoplasms, an aspect contrary to the reality presented in sinonasal tumors (17 , 45). These features, coupled with a disease-specific mortality of over 24%, underscore the aggressive nature of these neoplasms.

This review has important limitations. Most available evidence comes from case reports and small series, which are prone to bias, incomplete reporting, and heterogeneity in clinical and pathological details, limiting robust synthesis. Although we applied a comprehensive search strategy, restricting inclusion to English-language publications may have introduced language bias. The rarity of these tumors and the variability of available data also precluded subgroup or sensitivity analyses, leaving the certainty of evidence low. Many of these shortcomings are intrinsic to the study of extremely rare neoplasms, where evidence is necessarily fragmented. Moving forward, multicenter registries and collaborative studies are essential to consolidate cases, clarify molecular pathways, and identify therapeutic targets. Prospective evaluations of standardized multimodal regimens, including the role of elective neck dissection and molecularly guided therapies, are needed to improve outcomes in this aggressive disease.

## Conclusions

Non-sinonasal ITACs of the head and neck are exceptionally rare and aggressive tumors that predominantly affect the oral cavity, particularly the tongue, with a clinicopathological profile that mimics colorectal adenocarcinoma but displays distinct molecular alterations. Despite multimodal therapy, outcomes remain poor due to high rates of metastasis and disease-specific mortality. Accurate diagnosis requires integration of morphology with an extended immunohistochemical panel, while the lack of standardized treatment underscores the urgent need for multicenter registries and molecularly guided strategies to improve patient care.

## Figures and Tables

**Table 1:** Table Clinicopathological data extracted from published non-sinonasal intestinal-type adenocarcinomas of the head and neck.

Clinical data	n=37 (100%)
Gender	
· Male	27 (73%)
· Female	10 (27%)
Age - Decade of life	
· 3rd decade	2 (5.4%)
· 4th decade	3 (8.1%)
· 5th decade	4 (10.8%)
· 6th decade	11 (29.7%)
· 7th decade	11 (29.7%)
· 8th decade	4 (10.8%)
· 9th decade	2 (5.4%)
· Mean(±SD) - years	57.94(±14.50)
Location	
· Oral cavity	19 (51.4%)
o Mobile tongue	11 (29.7%)
o Palate	2 (5.4%)
o Buccal mucosa	2 (5.4%)
o Mouth floor	1 (2.7%)
o Mandible	1 (2.7%)
o Labial mucosa	1 (2.7%)
o Retromolar	1 (2.7%)
· Oropharynx (Tongue base)	7 (18.9%)
· Major salivary glands	4 (10.8%)
o Submandibular	3 (8.1%)
o Sublingual	1 (2.7%)
· Nasopharynx	2 (5.4%)
· Larynx	1 (2.7%)
· Middle ear (Eustachian tube)	1 (2.7%)
· Facial skin (Cheek)	1 (2.7%)
· Ectopic salivary gland	1 (2.7%)
· Hypopharynx	1 (2.7%)
Signs and symptoms	
· Swelling	25 (67.6%)
· Swallowing alterations	8 (21.6%)
· Pain	4 (10.8%)
· Breathing alterations	3 (8.1%)
· Ear/Hearing alterations	2 (5.4%)
· Phonation alterations	2 (5.4%)
· Bleeding	1 (2.7%)
· Not specified	12 (32.4%)
Evolution	
· Up to 1 month	1 (2.7%)
· >1 up to 3 months	6 (16.2%)
· >3 up to 6 months	7 (18.9%)
· >6 months up to 1 year	0 (0%)
· More than 1 year	2 (5.4%)
· Not specified	21 (56.8%)
· Mean(±SD) - months	34.31(±118.87)
Size	
· <1.0 cm	1 (2.7%)
· 1.0 up to 3.0 cm	9 (24.3%)
· 3.1 up to 5.0 cm	7 (18.9%)
· 5.1 up to 8.0 cm	3 (8.1%)
· 8.1 up to 10.0 cm	0 (0%)
· >10.0 cm	1 (2.7%)
· Not specified	16 (43.2%)
· Mean(±SD) - centimeters	3.99(±2.47)
Associated lymphadenopathy	
· Yes	13 (35.1%)
· No	10 (27%)
· Not specified	14 (37.8%)
Histopathological data	n=37 (100%)
Adenocarcinoma morphological patterns mentioned	
· Villous/Colonic	22 (59.5%)
· Mucinous	21 (56.8%)
· Papillary	10 (27%)
· Signet-ring cells	5 (13.5%)
· Cribriform	3 (8.1%)
· Not specified	3 (8.1%)
Tumor grading	
· Well-differentiated	1 (2.7%)
· Moderately differentiated	16 (45.9%)
· Poorly-differentiated	3 (8.1%)
· Not specified	17 (45.9%)
Other cytological/morphological features	
· Goblet cells	20 (54.1%)
· Infiltrative/invasive	20 (54.1%)
· Mitotic figures	18 (48.6%)
· Necrosis	16 (43.2%)
· Cytological atypia	17 (45.9%)
· Cystic/Multi-cystic areas	10 (27%)
· Perineural invasion	4 (10.8%)
· Lympho-vascular invasion	3 (8.1%)
· Squamous differentiation	1 (2.7%)
· Neuroendocrine differentiation	1 (2.7%)
Therapeutic and prognostic data	n=37 (100%)
Treatment	
· Surgery	9 (24.3%)
· Surgery + chemotherapy	2 (5.4%)
· Surgery + radiotherapy	7 (18.9%)
· Surgery + chemotherapy + radiotherapy	10 (27%)
· Surgery + chemotherapy + radiotherapy + immunotherapy	1 (2.7%)
· Chemotherapy + radiotherapy	4 (10.8%)
· Radiotherapy	2 (5.4%)
· Not performed	1 (2.7%)
· Not specified	1 (2.7%)
Local recurrence	
· Yes	3 (8.1%)
· No	25 (67.6%)
· Not specified	9 (24.3%)
· Recurrence time - Mean(±SD) - months	12(±0.0)
Regional and/or distant metastasis	
· Yes	13 (35.1%)
o Lymph node	12 (32.4%)
o Lung	6 (16.2%)
o Submandibular gland	2 (5.4%)
o Brain	1 (2.7%)
o Ovary	1 (2.7%)
o Skin	1 (2.7%)
· No	22 (59.5%)
· Not specified	2 (5.4%)
Prognostic status	
· Free of disease	20 (54.1%)
· Death from disease	9 (24.3%)
o Death time - Mean(±SD) - months	38.17(±45.47)
· Alive with disease	6 (16.2%)
· Not specified	2 (5.4%)
Follow-up - Mean(±SD) - months	46.12(±54.91)

SD: Standard deviation.

**Table 2:** Table Immunohistochemical data extracted from published non-sinonasal intestinal-type adenocarcinomas of the head and neck.

Citation	Case	CK7	CK20	CDX2	SATB2	Ki-67	Other positive antibodies	Other negative antibodies
[8]	23/M	-	+	ND	ND	85%	AE1/AE3, CK18, Î²-catenin	CK14, p63, TTF-1
[2] [5]	58/M	+/-	+	+	ND	ND	Î²-catenin, hMLH1, hMSH2, hMSH6, hPMS-2	-
	58/M	-	+	+	ND	ND	Î²-catenin, hMLH1, hMSH2, hMSH6, hPMS-2	-
[2,43]	58/F	+	-	-	ND	ND	CEA (+/-)	CK5/6, Napsin
[2] [44]	61/M	+	-	-	ND	ND	MUC1, EMA, Î²-catenin	CK5/6, CDX2, CK20, MUC2
[2]	5 cases	+	NS*	NS*	NS*	ND	-	-
[19]	31/M	-	+	+	+	ND	-	S100-P, AR, TTF-1/PAX8, GATA3, p16
	74/M	+	+	ND	+	ND	-	S100-P, AR, TTF-1/PAX8, p40/p63, GATA3, p16
[29]	55/M	+	+	ND	ND	ND	AE1/AE3, S100	-
[34]	63/M	-	+	ND	ND	ND	-	p63/p40, AR
[30]	35/M	+	-	-	ND	ND	SOX10, MUC1, MUC5A	S100, MUC2
	68/F	+	-	-	ND	ND	MUC1	SOX10, S100, MUC2, MUC5A
	63/M	+	+	-	ND	ND	MUC1, MUC5A	SOX10, S100, MUC2
	60/M	+	-	-	ND	ND	MUC1	SOX10, S100, MUC2, MUC5A
	59/F	+	-	-	ND	ND	MUC1	SOX10, S100, MUC2
	84/M	+	-	-	ND	ND	MUC1, MUC5A	SOX10, S100, MUC2
[20]	65/M	+	+	ND	ND	+	-	-
[7]	87/M	+	+/-	-	+	80%	AE1/AE3, CAM5.2, Î²-catenin, p53, CK14 (+/-), MUC2, MUC5 AC	p63, SMA, TTF-1, SP-A, CA19-9, PSA, Cyclin D1
[21] [46]	59/M	+/-	+	+	+	+	MUC2, MUC5A	Calponin, Her-2, p63, E-cadherin, PTEN
[22]	40/M	-	+	+	ND	ND	-	Napsin A, p40, TTF-1, p16
[31]	52/M	+/-	+	+	ND	60-90%	AE1/AE3, Synaptophysin, Chromogranin-A, CD56, CEA, p53	-
[23]	71/M	ND	+	+	ND	ND	CEA, AE1/AE3	-
[32]	54/M	+	+	+	ND	ND	AR, Î²-catenin, hMLH1, hMSH2, hMSH6, hPMS-2	TTF-1, p63, PSA
[24]	53/F	-	+	+	+	ND	AE1/3, Î²-catenin, hMLH1, hMSH2, hMSH6, hPMS-2	-
[26]	62/M	+	+	+	ND	ND	Pan-CK, CAM5.2, Villin, CEA, EMA, hMLH1, hMSH2, hMSH6, hPMS-2	TTF-1, MUC2, MUC5A, MUC6, p16, p63
[25]	63/F	+	-	+	ND	ND	Chromogranin (+/-), CK5/6 (+/-)	TTF-1
[44]	80/M	+	-	-	ND	ND	MUC-1, EMA, Î²-catenin	CK5/6, MUC2
[33]	60/M	+	+	+	ND	ND	-	TTF-1
[6]	49/M	+	+	+	ND	ND	AE1/AE3, CAM5.2, Villin, p16, CEA, EMA	-
[35]	41/M	-	+	ND	ND	ND	EMA, AE1/AE3, CEA	CK14, S100, TTF-1
[27]	69/M	+	+	+	ND	ND	CK18, MUC2, HEA125, Vimentin, HER-2, RC38, URO7	CK1, CK6, CK10, CK11, CK13, CK14, CEA, CA19-9, Thyroglobulin, MUC1, TTF-1
[28]	61/M	ND	ND	ND	ND	ND	-	SMA
[47]	67/M	ND	ND	ND	ND	ND	CAM5.2, CEA, EMA	-

ND: Not done; NS: Performed but not specified.

**Table 3:** Table Genetic data extracted from published non-sinonasal intestinal-type adenocarcinomas of the head and neck.

Citation	Case	Genomic features	Transcriptional features
[2]*	10 cases	MLL3, MAP3K4, TP53, and EGFR recurrent mutations	FOXA3, SPDEF, HNF1B, EpCAM, TOX3, and TNFSF11 upregulation
[30]	4/6 cases	AKT1 E17K hotspot mutations	-
[32]	54/M	Missense TP53 mutation (Leu194His); Wild-type KRAS, NRAS, BRAF and PIK3CA genes	-

*Refer to the article for the full results of the whole transcriptome and exome analysis.the head and neck.

## Data Availability

Declared none.
